# The Calcium Binding Protein S100A11 and Its Roles in Diseases

**DOI:** 10.3389/fcell.2021.693262

**Published:** 2021-06-11

**Authors:** Linqiang Zhang, Tingting Zhu, Huilai Miao, Bin Liang

**Affiliations:** ^1^Department of Hepatobiliary Surgery, Affiliated Hospital of Guangdong Medical University, Zhanjiang, China; ^2^Key Laboratory of Animal Models and Human Disease Mechanisms of the Chinese Academy of Sciences & Yunnan Province, Kunming Institute of Zoology, Chinese Academy of Sciences, Kunming, China; ^3^Department of Hepatobiliary Surgery, The Second Affiliated Hospital of Guangdong Medical University, Zhanjiang, China; ^4^Department of General Surgery, Dongguan Liaobu Hospital, Dongguan, China; ^5^Center for Life Sciences, School of Life Sciences, Yunnan University, Kunming, China

**Keywords:** S100 proteins, S100A11, diseases, signaling pathways, protein interaction

## Abstract

The calcium binding protein S100 family in humans contains 21 known members, with each possessing a molecular weight between 10 and 14 kDa. These proteins are characterized by a unique helix-loop-helix EF hand motif, and often form dimers and multimers. The S100 family mainly exists in vertebrates and exerts its biological functions both inside cells as a calcium sensor/binding protein, as well as outside cells. S100A11, a member of the S100 family, may mediate signal transduction in response to internal or external stimuli and it plays various roles in different diseases such as cancers, metabolic disease, neurological diseases, and vascular calcification. In addition, it can function as chemotactic agent in inflammatory disease. In this review, we first detail the discovery of S100 proteins and their structural features, and then specifically focus on the tissue and organ expression of S100A11. We also summarize its biological activities and roles in different disease and signaling pathways, providing an overview of S100A11 research thus far.

## Introduction

In 1965, in order to explore the unique proteins in the nervous system, American scientist Blake W. Moore, from Washington University School of Medicine, compared protein maps from brain and liver tissues of cattle. He found, through starch gel electrophoresis experiments, that one protein band migrated faster than the others, and that this protein band was only present in the brain tissue protein map (Moore, 1965; Moore and McGregor, 1965). After purification, it was found that this type of protein could be dissolved in a saturated ammonium sulfate solution, therefore this protein was named S100 protein. Subsequently, Blake W. Moore further confirmed that S100 protein was found in the brain tissues of 17 species including pigs, rats, mice, rabbits, hamsters, guinea pigs, dogs, monkeys, humans, turkeys, hawks, alligators, snakes, turtles, pompano fish, and red snapper. Data from rats demonstrated that the content of this protein in the brain is 1,000–10,000 times higher than in other tissues, hence the S100 protein was identified as a neural protein (Moore, 1965). Much later, it was shown that the S100 proteins identified by Moore were actually S100A1 and S100B, and that they were not only expressed in nervous tissue, but also were widely expressed in multiple tissues and cell types of vertebrates (Donato, 1999; Donato et al., 2009).

S100A11 is a member of the S100 protein family, and its cDNA cloning and protein purification was first done by Todoroki et al. (1991) in October, 1991. They reported that S100A11 has a molecular weight of 13 kDa, and possesses two EF-hand domains, which can bind calcium ions and undergo conformational changes. At the same time, they found the same protein in the aorta of cattle and the lungs of rabbits, and eventually named it calgizzarin. In December of the same year, Ohta et al. (1991) also isolated a protein with a molecular weight of about 11 kDa from the heart of pigs, and then cloned it. The cDNA was expressed *in vitro*, and subsequent amino acid sequence analysis showed that the protein had two EF-hand domains, with 40.9 and 37.5% homology with the known S100A1 and S100B proteins, respectively. The tissue expression pattern of this protein is apparently different from other known S100 proteins, which are highly expressed in lung and kidney tissues, but relatively low in liver and brain. Thus, they considered the protein to be a new S100 protein and named it S100C. Later, research found that calgizzarin and S100C are actually the same protein, and it is now collectively referred to as S100A11.

## The Family Members of S100 Proteins and Their Structures

**FIGURE 1:**
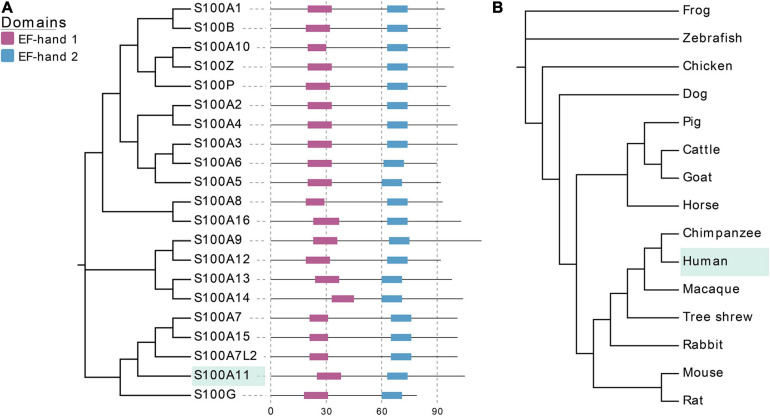
EF-hand domains of S100 family proteins and evolutionary relationships of S100A11 in different species. **(A)** The amino acid sequences of S100 proteins are from human, which can be found at UniProt (www.uniprot.org/). EF-hand 1 represents the non-canonical EF-hand, EF-hand 2 represents the canonical EF-hand. **(B)** The cladogram of CDS sequences of S100A11 gene in typical species of different families. The CDS sequences data are from NCBI (www.ncbi.nlm.nih.gov/).

At present, a total of 21 S100 proteins have been identified in humans (Bresnick et al., 2015), with their molecular weight varying between 10 and 14 kDa. There are also 21 corresponding genes that constitute the S100 gene family. 14 of the 21 genes are located in the q21 region of chromosome 1 (Eckert et al., 2004). S100 proteins exhibit high similarity with conserved EF-hand domains and share extensive structural homology (Figure 1A). Most S100 proteins exist as homo or heterodimers, and can bind calcium ions through their two EF-hand domains (Donato, 2003), in which one is a non-canonical EF-hand in the N-terminus and the other is a canonical EF-hand in the C-terminus, respectively (Figure 1A). Once bound to calcium ions, the conformation of the S100 proteins changes, which consequently makes them available to bind to targeting proteins and perform their corresponding biological functions. For example, they can play multiple roles in buffering calcium ion concentration (S100G) or sensing and transmitting calcium ion signals (other S100 proteins), they can also regulate enzyme activity, participate in energy metabolism, or regulate cell proliferation and differentiation (Donato et al., 2013). In addition, some S100 proteins can also be secreted outside the cell in an autocrine or paracrine manner, thereby acting as a signaling molecule to activate their corresponding receptors to participate in innate and acquired immune responses (Donato et al., 2013).

**FIGURE 2:**
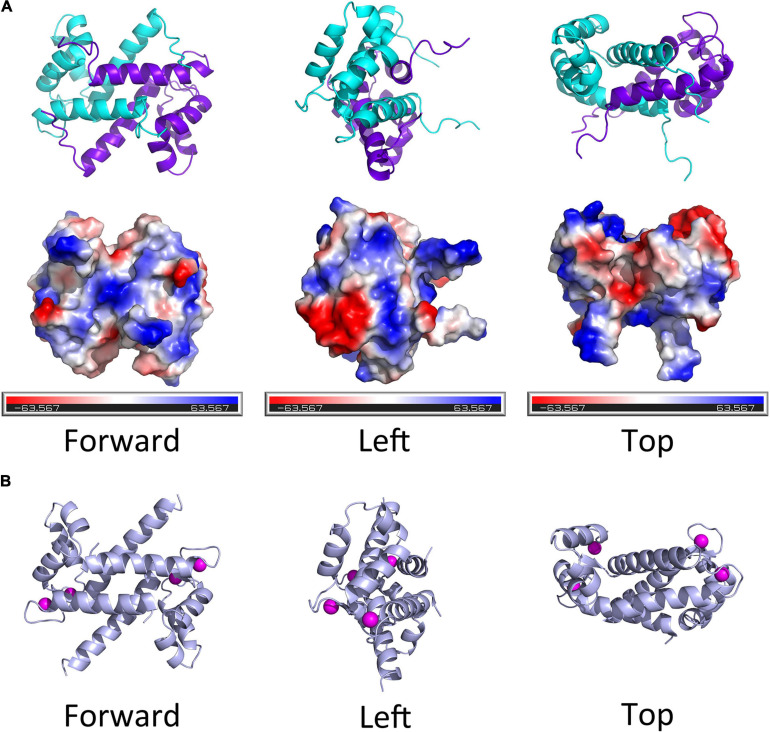
The diagram of spatial structure of S100A11 protein. **(A)** Top panel, the 3D structure of human S100A11 homodimer. Bottom panel, the surface charge profile of 3D structure of S100A11 homodimer shown in the same orientation as top panel, the surfaces with negative charge, positive charge and hydrophobic are colored in red, blue and white, respectively. The PDB file that used to make the 3D structure of human S100A11 was from RCSB PDB (www.rcsb.org/structure/2LUC), the 3D structures were made by PyMOL software. **(B)** The pig (*Sus scrofa*) S100A11 homodimer with the calcium ions. The calcium ions are shown as pink balls. The PDB file that used to make the 3D structure of porcine S100A11 was from RCSB PDB (www.rcsb.org/structure/1qls), the 3D structures were made by PyMOL software.

Thus far, there is no evidence for S100 proteins being present in invertebrates, though the S100 proteins are highly conserved among the vertebrates. Kraemer et al. (2008) studied the structural and functional diversification of S100 proteins in the teleost fish, which has the earliest S100 genes in terms of evolution. They found that several mammalian S100 genes have counterparts in teleost. Using S100A11 as an example, an evolutionary tree was ranked from *Osteichthyes* to *Amphibian*, *Aves*, and *Mammalia* (Figure 1B). A CDS sequence comparison of human S100 genes shows that *S100A11* has a higher sequence similarity with *S100G* and *S100A7L2* than the other family members (Figure 1A). Like several other members of the family, S100A11 can also form a homodimer (Figure 2A), which was verified by Réty et al. (2000) and Hung et al. (2012) in pig and in human, respectively. The structure of S100A11 homodimer with calcium ions (Réty et al., 2000) is shown in Figure 2B, which exhibits the locations of calcium ions in the S100A11 structure.

## Tissue and Cell-Type Specific Expression and Location of S100A11

**FIGURE 3:**
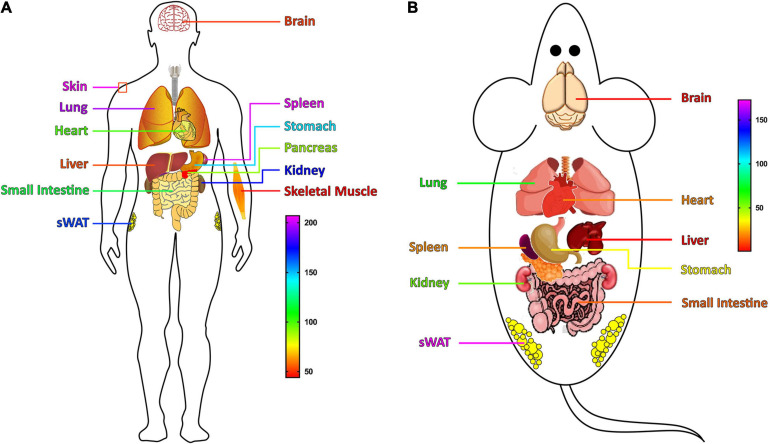
The tissues expression profile of the S100A11 gene. **(A)** The expression profile of the S100A11 gene in human tissues. S100A11 is highly expressed in human skin, spleen, and lung, and its expression is lower in liver, brain, and skeletal muscle. The expression data are from GeneCards (www.genecards.org/). **(B)** The expression profile of the S100A11 gene in mouse tissues. S100A11 is highly expressed in mouse sWAT, lung, and kidney, and it has a lower distribution in the small intestine, brain, and liver. The expression data are from NCBI (www.ncbi.nlm.nih.gov/). sWAT, subcutaneous white adipose tissue.

The expressions of S100A11, like other S100 proteins, is tissue and cell-type specific. In human, S100A11 is ubiquitously expressed in various tissues (He et al., 2009). In 1999, using Northern blot, Inada et al. (1999) showed that the expression level of *S100A11* is the highest in the placenta, followed by the heart, lungs, pancreas, and kidneys, and less in skeletal muscle, liver, and brain. However, following the development of sequencing technology, a more comprehensive tissue expression profile of S100A11 has been established. As shown in Figure 3A, human S100A11 is highly expressed in skin, spleen, lung, kidney, sWAT (subcutaneous white adipose tissue), and stomach; it is moderately expressed in the small intestine, heart, and pancreas; while it is less expressed in the liver, brain, and muscle. Likewise, in the mouse, S100A11 is highly expressed in the sWAT, lung, and kidney; it is moderately expressed in the stomach, spleen, heart, and small intestine; and it is less expressed in the brain and liver (Figure 3B).

The cellular distribution of S100A11 can vary in different tissues and it can also vary under distinct physiological conditions. For example, in the cells of normal kidney tissue, S100A11 is mainly distributed in the nucleus. In the cells of invasive cervical squamous cell carcinoma of the uterine cervix, S100A11 is distributed in both the nucleus and the cytoplasm. In cells of serous adenocarcinoma of the ovary, S100A11 is mainly distributed in the nucleus, with only a small amount in the cytoplasm. However, in the cells of invasive breast carcinoma of ductal no special type, S100A11 shows a strong distribution in the cytoplasm, with only a weak amount in the nucleus (Cross et al., 2005).

The dynamic distribution of S100A11 in cells also has been investigated. In human keratinocytes, S100A11 is uniformly distributed in the cytoplasm under normal conditions, but when stimulated with a high concentration of calcium ions (0.3 mmol/L), it interacts with another protein, Annexin I, to accumulate on the inner membrane side of the cell membrane with the help of tubulin. This process does not rely on the classical Golgi/ER export pathway. Through subsequent exosome generation at the plasma membrane, S100A11 can be secreted from the cell. Now extracellularly localized, S100A11 can act as a signal molecule to bind the RAGE (receptor for advanced glycation end products) receptor (Broome and Eckert, 2004). Overall however, based on the sum of this data, we can summarize that S100A11 can be distributed in the nucleus, cytoplasmic matrix, and extracellular region, which is cell type and tissue state dependent.

## The Biological Activities of S100A11

**FIGURE 4:**
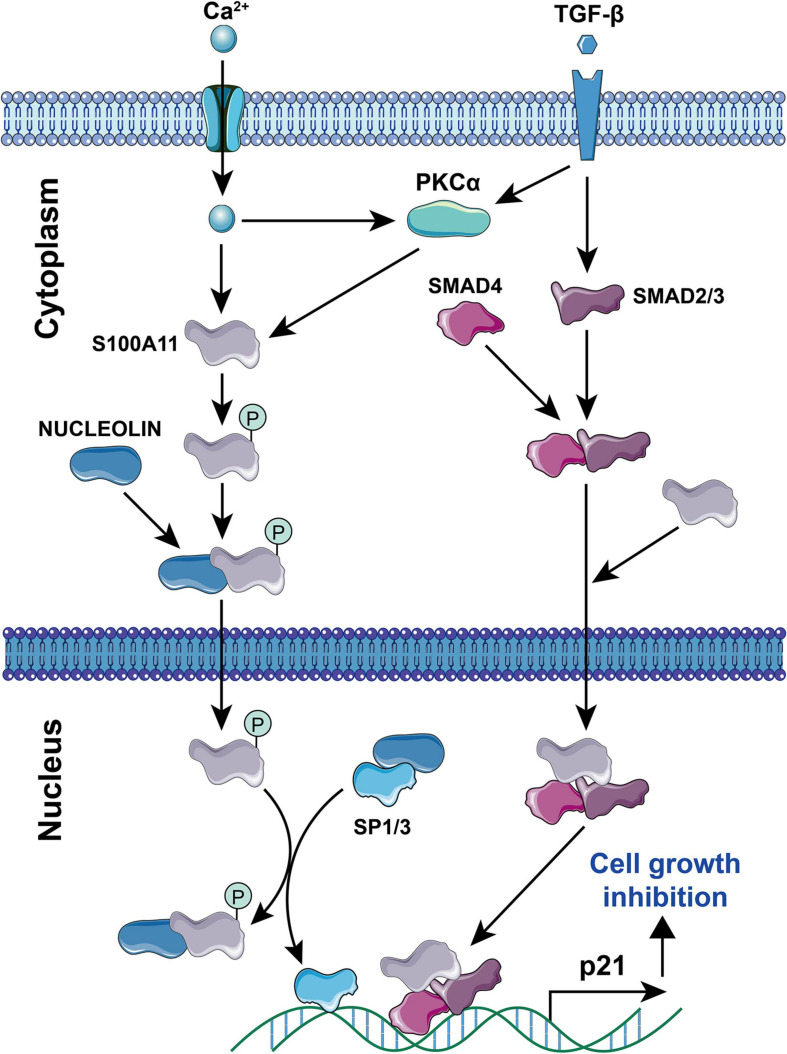
Signaling pathways for Ca^2+^ and TGF-β-induced S100A11-mediated cell growth inhibition. Elemental Ca^2+^, or PKCα induced by Ca^2+^/TGF-β can promote the phosphorylation of S100A11. Phosphorylated S100A11 then translocates to the nucleus through binding to NUCLEOLIN. In the nucleus, S100A11 competes with Sp1/3 for binding to NUCLEOLIN. This then releases free Sp1/3 to induce p21 expression. Moreover, S100A11 can bind to SMAD2/3 and SMAD4, that has been stimulated by TGF-β, to form a complex. Subsequently, this complex migrates to the nucleus to induce p21 expression. In either case, increasing levels of p21 results in cell growth inhibition. The circled “P” represents phosphorylation.

**FIGURE 5:**
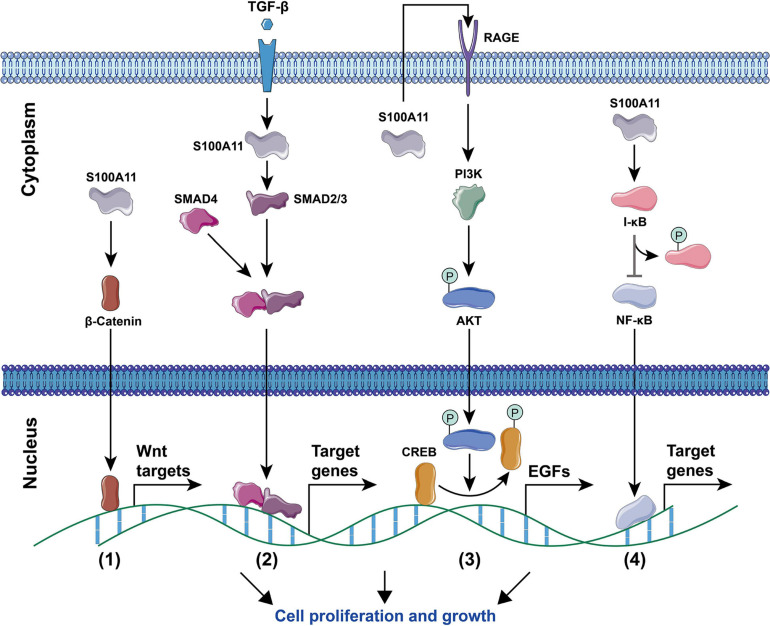
Signaling pathways for S100A11-mediated cell proliferation and growth. **(1)** The Wnt signaling pathway. S100A11 accelerates the entry of β-Catenin into the nucleus, thereby regulating the downstream Wnt target genes. **(2)** The TGF-β signaling pathway. Under the stimulation of TGF-β, S100A11 promotes the SMAD2/3 to form a complex with SMAD4, the complex then enters into the nucleus and controls the expression of target genes. **(3)** The PI3K/AKT signaling pathway. S100A11 is first secreted outside the cell and then binds to the RAGE receptor to induce the expression of EGF genes through the Akt signaling pathway. CREB, cAMP response element-binding protein; EGFs, epidermal growth factors. **(4)** The NF-κB signaling pathway. S100A11 promotes the phosphorylation of I-κB, which then leads to the activation and nuclear migration of NF-κB to regulate the expression of target genes. All of the above four processes ultimately promote cell proliferation and growth. The circled “P” represents phosphorylation.

**FIGURE 6:**
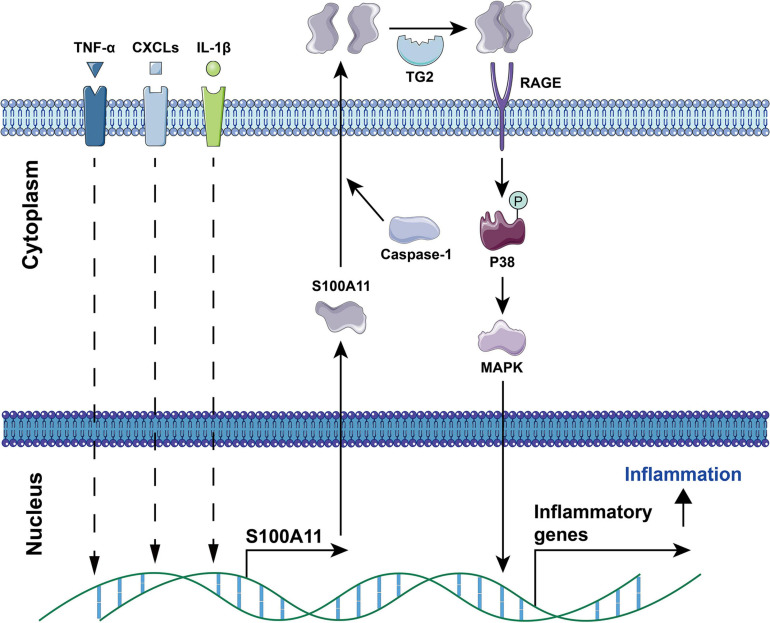
The involvement of S100A11 in TNF-α, CXCLs, and IL-1β induced inflammation. TNF-α, CXCL8, and IL-1β induce the expression of S100A11. Subsequently, S100A11 is secreted through a Caspase-1 dependent manner. Extracellular S100A11 is dimerized under the catalysis of TG2, and then binds to the RAGE receptor to induce the expression of inflammatory genes through the p38 MAPK signaling pathway, which causes inflammation progression. CXCLs, CXC chemokine ligands; TG2, transglutaminase 2. The circled “P” represents phosphorylation.

Current studies have found that S100A11 has the following main functions: (1) Regulation of enzyme activity. In smooth muscle, S100A11 can interact with actin filaments and inhibit ATPase activity in the presence of calcium ions (Zhao et al., 2000). (2) Regulation of cell growth. Using human keratinocytes as example, when the cells are stimulated by calcium ion or TGFβ, S100A11 will be phosphorylated by PKCα and then enter the nucleus to promote the expression of the *p21* gene, which further inhibits keratinocyte proliferation by inhibiting *cdk2* (Sakaguchi et al., 2003, 2004), this process is illustrated in Figure 4. Conversely, down-regulation of S100A11 can lead to a decrease in the protein level of p21. It has been shown that the down-regulation of S100A11 caused a decrease in phosphorylation of AKT, which then activated GSK3. Activated GSK3 can phosphorylate threonine at position 57 in p21, which causes the degradation of p21 protein in a ubiquitination-independent manner. Therefore, the down-regulation of S100A11 promotes the turnover of p21 through the PI3K/AKT signaling pathway, thereby promoting the cell proliferation process (Foertsch et al., 2013). In addition, other pathways that are involved in the regulation of cell proliferation and growth by S100A11 are summarized in Figure 5. (3) Inducing apoptosis. Studies in human tumor cell lines, such as melanoma, pancreatic cancer, breast cancer, and lung cancer, have found that a 19 amino acid peptide from the N-terminus of the S100A11 protein can promote the translocation of apoptosis-inducing factor (AIF) from the cytoplasm to the nucleus. AIF in the nucleus triggers chromatin condensation and DNA fragmentation, thereby inducing apoptosis of tumor cells (Makino et al., 2004). (4) Participating in the inflammatory response (Figure 6). During the occurrence of osteoarthritis, CXCL8 (C-X-C Motif Chemokine Ligand 8) and TNF-α (Tumor necrosis factor α) can induce the expression of S100A11 and promote its release to the extracellular space to form a dimer. This dimer then binds to the receptor RAGE, promoting the development of osteoarthritis through the p38 signaling pathway (Cecil et al., 2005; He et al., 2009).

S100A11 was also shown to have a role in the organization of early endosomes. Seemann et al. (1997) reported that the deletion of 14 amino acid residues in the C-terminal of the S100A11 protein abolished the interaction between S100A11 and Annexin A1 (ANXA1), which then restricted the endosomal localization of this complex and restrained the structural organization of the endosome membranes. Since ANXA1 has been linked to the internal vesiculation process and structural organization in multivesicular endosomes by localizing on the membranes, binding with S100A11 could modulate the physiological properties of ANXA1 through phosphorylation by protein kinase C (Fava and Cohen, 1984; Futter et al., 1993; Seemann et al., 1996). Thus, physiologically, this complex could possibly hold the membranes in place for endosome fusion to occur or could organize the compartment of endosome. In addition, the interaction of S100A11 with ANXA1 promotes the transport of cholesterol from the endoplasmic reticulum to the endosome by mediating membrane contact between these two organelles. The transport of cholesterol is a process that is necessary for the formation of intraluminal vesicles within the endosome, which then can regulate the degradation of the epidermal growth factor receptor (EGFR). Therefore, S100A11 also plays an important role in the regulation of EGFR signaling (Ringerike et al., 2002; Eden et al., 2016; Tan et al., 2016). Moreover, S100A11 was shown to form a complex with ANXA1 to enhance lysosomal targeting of the EGFR, which then led to interruption of EGF signal and degradation of EGFR. In this case, the complex functions as a physical scaffold to support the sorting of EGFR into multivesicular bodies (Poeter et al., 2013).

## The Roles of S100A11 in Diseases

Dysregulation of S100A11 has been shown to be involved in various human diseases *via* different signaling pathways. A summation of these results is detailed in Table 1. In addition, several studies have shown that S100A11 can form a complex with specific proteins (Table 2) in some diseases. Saho et al. (2016) showed that in mesothelioma cells, S100A11, lacking the classical secretory signal, can be dimerized and secreted through the peroxisome and thus play a key role in mesothelioma progression in a tumor microenvironment. Moreover, extracellular vesicles (EVs), such as exosomes and microvesicles (Raposo and Stoorvogel, 2013), have been shown to display marked potential in the organ-to-organ communications (Thomou et al., 2017). A recent study reported that in both human and mouse, cholangiocyte cell lines could release EVs containing S100A11 to induce bone marrow-derived macrophages to express pro-inflammatory cytokines (Katsumi et al., 2019).

**TABLE 1:** Pathways that S100A11 is associated with in differentdiseases and cell types.

**Pathways**	**Diseases or cell types**	**References**
AMPK signaling	Arterial neointima	Yu et al., 2012
EGFRvIII-STAT3 pathway	Hepatocellular carcinoma	Luo et al., 2013
EGFR signaling	HeLa and A431 cells	Poeter et al., 2013
PI3K/AKT signaling pathway	Hypopharygeal squamous cell carcinoma Pancreatic cancer Human keratinocyte HaCaT cells	Wang et al., 2019 [Bibr B88][Bibr B24]
P38/MAPK pathway	Intrahepatic cholangiocarcinoma Osteoarthritis	[Bibr B93][Bibr B7]; Cecil et al., 2009
NF-κB pathway	Biliary tract diseases Glioblastoma	Katsumi et al., 2019 Tu et al., 2019
TGF-β1/SMAD pathway	Intrahepatic cholangiocarcinoma Pancreatic cancer Colorectal cancer	[Bibr B94][Bibr B35] Niu et al., 2016
TGF-β signaling	Human keratinocytes	Sakaguchi et al., 2004
Wnt/β-Catenin Signaling	Cervical squamous cell carcinoma	Meng et al., 2019

**TABLE 2:** Proteins that can interact with S100A11.

**Proteins**	**Functions of the complex**	**References**
ANXA1	Keratinocyte activation Organization of endosomes Promotes ER to endosome cholesterol transport	Dowarha et al., 2018 Seemann et al., 1997 Eden et al., 2016
ANXA2	Plasma membrane repair in cancer cells Hepatocellular carcinoma development Promotes the progression of glioblastoma	Jaiswal et al., 2014; [Bibr B40][Bibr B74] Tu et al., 2019
ANXA6	Ca^2+^-dependent linkage of the plasma membrane to the cytoskeleton	Chang et al., 2007
HDAC6	Reduce the deacetylation of FOXO1 in hepatocyte	Zhang et al., 2021
PEX14	Promotes the peroxisomal secretion of dimerized S100A11	Saho et al., 2016
P53	Regulation of tumor growth	Fernandez-Fernandez et al., 2008
RAD51	DNA double-strand breaks (DSBs) repair	Foertsch et al., 2016
RAD54B	DNA double-strand breaks (DSBs) repair	Murzik et al., 2008
RAGE	Inflammation-induced chondrocyte hypertrophy Mediator forgrowth regulation of human keratinocytes	Cecil et al., 2005 Sakaguchi et al., 2008

### Cancers

S100A11 is involved in many types of cancers and it plays a distinct role depending on the specific tumor type. A summation of these results is detailed in Table 3. In most cancers, S100A11 is highly expressed and correlated to tumor promotion and progression. Studies have found that in renal cell carcinoma, which is prevalent in North America, the expression level of S100A11 is positively correlated to the degree of cancer progression and tumor size, and negatively correlated to the disease-free survival rate of patients (Gabril et al., 2016). In addition, in prostate and breast cancer, the expression of S100A11 is significantly associated with high pathologic cancer stage, suggesting that S100A11 is involved in cancer development and progression (Rehman et al., 2004; Cross et al., 2005). Sobolewski et al. (2020) found that overexpression of S100A11 leads to high-grade hepatocellular carcinoma and poor prognosis by promoting cancer cell proliferation and migration. They therefore suggested that S100A11 be considered as an oncogenic factor (Sobolewski et al., 2020). In these cases, the up-regulation of S100A11 significantly promotes the proliferation, migration, and tissue invasion of tumor cells by activating different signal pathways (Meng et al., 2019; Mitsui et al., 2019). A S100A11/ANXA2 complex has also been shown to be able to reseal the plasma membrane if it has become damaged due to the stress of metastatic tissue invasion (Jaiswal et al., 2014; Sobolewski et al., 2020).

**TABLE 3:** S100A11 expression in different cancers.

**Cancers**	**Expression level**	**References**
Bladder cancer	Down-regulated	Memon et al., 2005
Esophageal squamous cell carcinoma	Down-regulated	Ji et al., 2004
Breast cancer	Up-regulated	Cross et al., 2005
Cervical squamous cell carcinoma	Up-regulated	Meng et al., 2019
Clear cell sarcoma of soft tissue	Up-regulated	Schaefer et al., 2004
Colorectal carcinoma	Up-regulated	Tanaka et al., 1995; Stulik et al., 1999; Guo et al., 2021
Gastric carcinoma	Up-regulated	Oue et al., 2004
Glioblastoma	Up-regulated	Tu et al., 2019
Hepatocellular carcinoma	Up-regulated	Jiang et al., 2019; Sobolewski et al., 2020
Lung adenocarcinomas	Up-regulated	Woo et al., 2015
Ovarian cancer	Up-regulated	Liu et al., 2015
Pancreatic carcinoma	Up-regulated	Ohuchida et al., 2006; Mitsui et al., 2019
Prostate cancer	Up-regulated	Rehman et al., 2004
Renal cell carcinoma	Up-regulated	Gabril et al., 2016
Thyroid carcinoma	Up-regulated	Torres-Cabala et al., 2004; Anania et al., 2013
Uterine smooth muscle tumors	Up-regulated	Kanamori et al., 2004

In tumors with a high expression of S100A11, such as in intrahepatic cholangiocarcinoma, the silencing of S100A11 can inhibit TGF-β1-induced cell migration, invasion, and epithelial-mesenchymal transition (EMT). Upon silencing of S100A11, the phosphorylation levels of SMAD2/3, induced by TGF-β, also are decreased. Therefore, conversely, one of the reasons for the invasivity of this type of tumor may be that S100A11 induces phosphorylation of SMAD2/3 through TGF-β, which then accelerates the process of tumor cell migration, tissue infiltration, and EMT (Zhang et al., 2018).

On the other hand, in bladder cancer, S100A11 is thought to function as a tumor suppressor. Memon et al. (2005) found that the mRNA and protein levels of S100A11 were all down-regulated in patients with bladder cancer. In addition, a negative correlation between S100A11 expression and bladder cancer progression was observed, and loss of S100A11 was associated with poor survival in bladder cancer patients (Memon et al., 2005). At the same time, research in fibroblasts found that when normal cells shift to an immortal state, the expression level of S100A11 is also reduced (Sakaguchi et al., 2000). The underlying mechanism may be related to the function of S100A11 being able to be phosphorylated, with the phosphorylated S100A11 entering the nucleus and inducing the p16 and p21 proteins to inhibit cell DNA synthesis (Sakaguchi et al., 2000, 2001). Taken altogether, these studies suggest that the level of S100A11 expression, the signaling pathways that S100A11 is involved in, and the posttranscriptional modification status of S100A11 protein contribute to the different roles of S100A11 in different tumors.

Furthermore, in a recent study on human cancers, S100A11 was found to be highly enriched in the proteomic profile of extracellular vesicles and particles (EVPs) in human pancreatic adenocarcinoma. It was identified as a tumor-derived EVP protein involved in eliciting immune responses, suggesting that S100A11 is also involved in tumor immunity (Hoshino et al., 2020).

### Metabolic Disease

Type 2 diabetes (T2D) is an increasing global health problem, and the gene regulation in human pancreatic islets under T2D is complicated. Using global genomic and transcriptomic analysis of human pancreatic islets from 89 donors, Fadista et al. (2014) found that *S100A11* positively associated with HbA1c. HbA1c is one of the genes that can influence glucose metabolism, and it plays an important role in the pathogenesis of T2D (Fadista et al., 2014). In addition to T2D, non-alcoholic fatty liver disease (NAFLD) is another metabolic disease mainly induced by high energy consumption, for example a high fat or high fructose diet. Zhou et al. (2017) found that in a mouse NAFLD model generated by a western diet (40% fat and 0.2% cholesterol) treatment, the expression of S100A11 was significantly upregulated in the liver. Another study using a mouse model of non-alcoholic steatohepatitis treated with a high fat/high cholesterol diet (20% fat and 1% cholesterol) showed that S100A11 increased in the liver in a time-dependent manner (Oh et al., 2013).

**FIGURE 7:**
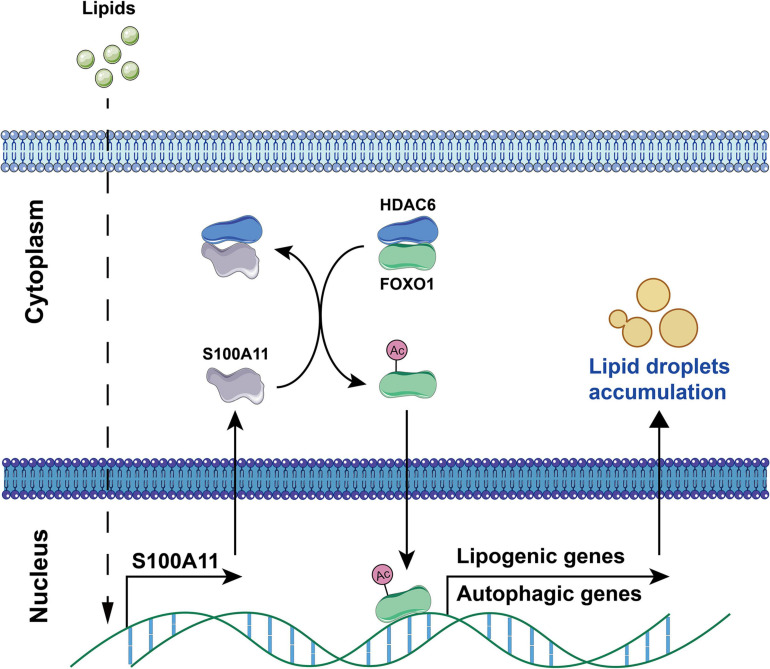
S100A11-HDAC6-FOXO1 axis in the regulation of autophagy and lipogenesis in liver induced by high fat diet. Dietary lipids induce the overexpression of S100A11. S100A11 then competes with FOXO1 for binding to HDAC6, which releases and promotes the acetylation of FOXO1. The acetylated FOXO1 activates autophagy and lipogenesis pathways leading to lipid droplet accumulation. HDAC6, histone deacetylase 6; FOXO1, forkhead box protein O1. The circled “Ac” represents acetylation.

According to the latest study in our lab (Zhang et al., 2021), it was confirmed that S100A11 was significantly upregulated in the liver of a tree shrew NAFLD model (Zhang et al., 2015, 2016). Following this result, we overexpressed S100A11 in *Hepa 1-6* and human *Hep 3B* cell lines and found that overexpression resulted in enhanced lipid(s) accumulation. More importantly, overexpression of S100A11 in mice liver also accelerated the hepatic lipids deposition. Mechanistically, our results indicate that exogenous dietary lipids promote liver S100A11 expression, which then competitively interacts with HDAC6 to block the binding between HDAC6 and FOXO1. Loss of HDAC6 then promotes the acetylation of FOXO1, consequently activating autophagy and lipogenesis pathways, thereby accelerating liver lipid accumulation (Figure 7). Coincidentally, Teng et al. (2021) also reported that S100A11 could contribute to hepatic steatosis through RAGE-mediated AKT-mTOR signaling. In this study the experimental phenotype is consistent with ours, but the hypothesized molecular mechanism is different (Teng et al., 2021). In any case, these studies do show that S100A11 can affect autophagy and lipid metabolism, and that it plays an important and complex role in fatty liver disease.

Although there are relatively few studies on S100A11 in metabolic diseases, these published works indicate that S100A11 may play an important role in the development and progression of other metabolic diseases, not only T2D and NAFLD.

### Neurological Diseases

S100A11 has also been shown to be an important factor in neurological diseases. Amyotrophic lateral sclerosis (ALS) is a neurodegenerative disease characterized by a degeneration of motor neurons in the spinal cord and motor cortex. Studies have shown that S100A11 was significantly enriched in ALS where it functions as a calcium sensor and modulator to promote vulnerability to neurodegeneration (Alexianu et al., 1994; Iridoy et al., 2018). In another severe neurological disease, autoimmune encephalitis, S100A11 was also found to be up-regulated and hypomethylated. Along with S100A6, in this state specific S100 family members may facilitate B lymphocyte infiltration into the central nervous system through the blood-brain barrier (Tsai et al., 2019). However, it has been suggested that S100A11 does not always play a role in pathogenicity. Ischemia-reperfusion can directly lead to cell death. This can subsequently result in many neurological conditions. ANXA1 nuclear translocation has long been recognized as a pathogenic factor through inducing neuronal apoptosis (Li et al., 2016). In a mouse model of ischemic stroke, S100A11 exerts neuroprotective effects, and upregulation of S100A11 protected against neuronal apoptosis by interaction with ANXA1 through the nuclear translocation signal (NTS) of ANXA1, thereby blocking ANXA1 nuclear translocation (Xia et al., 2018). According to these studies, like its role in tumors, S100A11 can also have a dual role in neurological diseases.

### Vascular Calcification

Vascular calcification is a highly prevalent vascular pathophenotype that has been associated with aging, atherothrombotic cardiovascular disease, diabetes mellitus, and chronic kidney disease (Leopold, 2015). In a mouse model of chronic kidney disease, S100A11 and its receptor, RAGE (receptor for advanced glycation end products), were both up-regulated. S100A11/RAGE signaling activates ERK1/2 MAPK, which further activates mTORC1 and the ER stress-Unfolded Protein Response to induce vascular calcification (Panda et al., 2018). Coincidentally, in the ectonucleotide pyrophosphatase/phosphodiesterase 1 knockout (*Enpp1*^–/–^) mice model of arterial calcification, it was shown that S100A11 was also highly expressed in the aorta. Mechanistically, dependent upon RAGE, S100A11 induced cartilage-specific collagen IX/XI expression to promote ectopic chondrogenic differentiation and calcification in the aorta (Cecil and Terkeltaub, 2011). In addition to accelerated calcification, S100A11 also plays a role in neointima formation in response to arterial injury. S100A11 positive cells were significantly enriched in injured arteries. Furthermore, S100A11/RAGE signaling mediates vascular remodeling by regulation of the AMPK pathway *via* liver kinase B1 and STAT3 (Yu et al., 2012). Thus, what we know about the function of S100A11 in vascular calcification is largely dependent on its receptor, RAGE.

### Inflammatory Diseases

In osteoarthritis, inflammation promoted chondrocyte differentiation always contributes to the disease progression. Studies have shown that S100A11 can be released by the chondrocyte after stimulation by IL-1β. Once secreted, S100A11 is then covalently crosslinked into a dimer through the catalysis of transglutaminase 2 (Cecil and Terkeltaub, 2008). The dimerized S100A11 acquires the capacity to promote chondrocyte activation through the p38/MAPK pathway, which then accelerates the development of osteoarthritis (Cecil et al., 2005; Cecil and Terkeltaub, 2008; Figure 6). By contrast, rheumatoid arthritis is a chronic systemic autoimmune disease. Andres Cerezo et al. (2017) found that rheumatoid arthritis patients have increased levels of S100A11 in their synovial tissue and synovial fluid but not in serum. The increased amount of S100A11 can stimulate the production of the pro-inflammatory cytokine IL-6 by peripheral blood mononuclear cells and synovial fibroblasts, suggesting an association between S100A11, inflammation, and disease activity in rheumatoid arthritis patients (Andres Cerezo et al., 2017). Further, a recent study (Navrátilová et al., 2021) uncovered that, under rheumatoid arthritis conditions, S100A11 could be released by neutrophils. The extracellular S100A11 then enhanced the secretion of IL-6 and TNF by neutrophils and subsequently aggravated the inflammatory response.

S100A11 also has functions in other inflammatory diseases. CCL2 is a crucial chemokine required for host resistance to parasites. It has been recently reported that after infection by *Toxoplasma gondii*, human monocytes detect S100A11 protein, which has been released from infected cells in a caspase-1-dependent manner. S100A11 binding to RAGE induces the production of the chemokine CCL2 to resist further *Toxoplasma gondii* infection. Therefore S100A11 plays a role of innate immune sensor in this infection (Safronova et al., 2019). In addition, studies have shown that circulating S100A11 can be used as a biomarker of myositis (Andrés Cerezo et al., 2019) and infective endocarditis (Thuny et al., 2012). While all these studies give strong evidence for the importance of S100A11 in inflammatory diseases, further research is necessary to determine if this would be a useful clinical target to ameliorate inflammation.

## Future Perspectives and Challenges

The S100 protein family constitutes the largest subgroup of the EF-hand family of Ca^2+^-binding proteins, and S100A11 is one of these proteins. To date, a large number of studies have shown that S100A11 has biological functions such as regulating cell growth, enzyme activity, and the inflammatory response. As well, it is involved in the regulatory process of cancers, metabolic diseases, neurological diseases, vascular calcification, and inflammatory diseases. Taken together, all the evidences cited in this review indicate that S100A11 is an important molecule involved in many diverse aspects of cellular functions.

The studies show that S100A11 is involved in many different biological processes, for which we speculate that this may be related to the characteristics of S100A11. First, as a protein that can bind calcium ions, S100A11 may perform distinct functions due to binding with or without calcium ions. Secondly, S100A11 is a protein with small molecular weight that can be secreted extracellularly and can also be transported in the blood by means of exosomes, extracellular vesicles, or as a covalently bound dimer. Thus, it may be involved in the cross-talk among and between cells as well as organs. This may also account for its functional diversity. Ultimately, as a protein that can enter the nucleus, S100A11 can directly or indirectly affect the expression of different genes and thus exert various functions.

As far as the current research is concerned, our understanding of S100A11 is very limited, and there are still many challenges to be overcome in the future. First of all, it is not clear how S100A11 is regulated at the level of transcription, translation, or the status of post-translational modification(s) under physiological and/or pathological conditions. Secondly, although several studies have shown that S100A11 is involved in certain signaling pathways and can form complexes with proteins in some diseases, nevertheless, the specific molecular mechanism of S100A11 function in these diseases is still not clear. Thirdly, most of the studies on S100A11 are mainly focused on cancers, such as breast cancer or colorectal cancer, implying that S100A11 is expected to be developed as a biomarker or therapeutic target for tumors. However, S100A11 plays a dual opposing role for tumor promotion and tumor inhibition in the progress of different cancers, while, again, the underlying molecular mechanisms are still unknown. Clearly, further research is needed to study the dichotomy of S100A11 in different cancers. Furthermore, S100A11 has long been regarded as a secretory protein and biomarker for some cancers. Whether S100A11 is always secreted in the form of extracellular vesicle cargo in different organs and diseases, and if during this process whether the S100A11 protein is modified after secretion is unknown. Fourthly, S100A11 has a clear functionality in osteoarthritis. However, many questions concerning its functionality have not yet been answered, such as the pathway through which inflammatory factors (TNF-α, CXCLs, and IL-1β) induce the expression of S100A11 and release it from cartilage, the region where S100A11 interacts with RAGE, and ultimately how the signal is transduced to activate the P38 phosphorylation pathway. Finally, there is very little research on S100A11 in other areas, especially in metabolic diseases, which are widely prevalent worldwide. Therefore, the role of S100A11 in metabolic diseases deserves much greater consideration in the future.

The application of modern biology and bioinformatics tools to study the mechanistic and functional interaction between S100A11 and its related proteins, as well as deciphering its intrinsic cell signal transduction pathways in-depth in multiple cell types, will contribute to the clinical diagnosis, prevention, and treatment of S100A11 related diseases in the future.

## Author Contributions

LZ conceived and wrote the original draft. TZ collected data of the CDS sequences and amino acid sequences of S100 proteins as well as the data of expression profile of human and mouse *S100A11* genes. HM provided guidance for the draft. BL provided advice and supervision, and revised the manuscript. All the authors contributed to the article and approved the submitted version.

## Conflict of Interest

The authors declare that the research was conducted in the absence of any commercial or financial relationships that could be construed as a potential conflict of interest.
